# Light-Sheet Microscopy for Surface Topography Measurements and Quantitative Analysis

**DOI:** 10.3390/s20102842

**Published:** 2020-05-16

**Authors:** Zhanpeng Xu, Erik Forsberg, Yang Guo, Fuhong Cai, Sailing He

**Affiliations:** 1 Centre for Optical and Electromagnetic Research, National Engineering Research Center for Optical Instruments, Zhejiang Provincial Key Laboratory for Sensing Technologies, College of Optical Science and Engineering, Zhejiang University, Hangzhou 310058, China; zhanpengxu@zju.edu.cn (Z.X.); erikf@zju.edu.cn (E.F.); guo.yang@zju.edu.cn (Y.G.); 2 School of Biomedical Engineering, Hainan University, Haikou 570228, China; caifuhong@zju.edu.cn

**Keywords:** light-sheet microscopy (LSM), laser triangulation, surface topography, line scanning, 3D reconstruction, quantitative analysis

## Abstract

A novel light-sheet microscopy (LSM) system that uses the laser triangulation method to quantitatively calculate and analyze the surface topography of opaque samples is discussed. A spatial resolution of at least 10 μm in *z*-direction, 10 μm in *x*-direction and 25 μm in *y*-direction with a large field-of-view (FOV) is achieved. A set of sample measurements that verify the system′s functionality in various applications are presented. The system has a simple mechanical structure, such that the spatial resolution is easily improved by replacement of the objective, and a linear calibration formula, which enables convenient system calibration. As implemented, the system has strong potential for, e.g., industrial sample line inspections, however, since the method utilizes reflected/scattered light, it also has the potential for three-dimensional analysis of translucent and layered structures.

## 1. Introduction

Micro-optical imaging technology is noninvasive, offers high speed and high resolution, and is used extensively in biological research as well as for industrial applications. In traditional microscopic imaging, attention is on the 2D morphological characteristics of objects, however, it is impossible to acquire the full spatial information of an object with 2D image information. As a consequence, 3D measurements have become a commonplace inspection method for, e.g., textured steel sheets, silicon wafers and metallic surfaces [1,2,3].

Mechanical stylus measurements [4,5] have been used in recent years for 3D sample surface topography inspection, however, they easily damage the surface of the sample. To avoid this drawback, several non-contact optical technologies that construct 3D spatial models through microscopic imaging have been proposed. For example, laser-scanning confocal microscopy [6,7] illuminates a point which is confocal, with the pinhole in front of the photodetector; multiphoton microscopy [8] excites the point in focus; structured light projects the surface for shape measurement [9]; and stereo vision [10,11] captures multi-angle images for inspection without active illumination. Point-scanning microscopy techniques are, however, limited by 2D movements, causing low imaging efficiency. Structured illumination-based optical sectioning microscopy is highly susceptible to sample motions [12]. For stereo vision, the working distance and the size of the objective restricts its use in multi-perspective microscopic 3D imaging.

Compared to traditional contact and non-contact methods, line scan detection technology is able to cover an entire plane with a single scan and has the advantages of being non-contact, having high efficiency, low phototoxicity and low photobleaching, as well as being able to maintain spatial and spectral resolutions throughout the measurement process [13,14]. As a consequence, the technology is widely used for, e.g., bio-imaging, food detection and industrial measurements [15,16,17]. Light-sheet microscopy (LSM) was first reported by Huisken et al. [18] for in-vivo bio-imaging that demonstrated the method′s high contrast and axial resolution, low photobleaching and low photo damage. Subsequently, a series of light-sheet microscopy technologies, such as digital scanning light microscopy (DSLM) [19,20], light-sheet fluorescence microscopy (LSFM) [21,22,23] and multidirectional LSM [24,25,26], have been developed. Most of these LSM systems have been used for penetration imaging or fluorescence imaging of biological samples, and they typically have long imaging times (e.g., in the order of 10 min [22,23]). It is worth mentioning that a few companies, such as Keyence or NEO Subsea AS, have launched commercial sensing systems using light-sheet technology for high-speed measurements of 3D surface topographies of non-transparent samples. However, the compact mechanical structure used limits the layout of an optical path, necessitating a more complex and time-consuming calibration relationship. The fixed commercial structure designs also mean that the resolution or field-of-view (FOV) cannot be adjusted to adapt to different applications by replacing some components.

We have previously proposed a light-sheet microscopy setup for qualitative 3D detection without distortion [27] by utilizing reflected or backscattered light. The system was however not able to analyze surface variations in a quantitative way and the manual aspects make the method relatively inefficient. In this paper, we present a high-speed, low-cost LSM inspection system that includes quantitative analysis of sample surface variations. The system is based on line-scanning in which an illumination unit generates a thin light-sheet that selectively illuminates the surface of the sample, and layer-by-layer scanning of the sample is realized by the movement of a displacement stage. The sample surface topography will modulate the light-sheet in the height direction and, based on this, we can construct a 3D image of the sample′s surface topography without distortion. By integrating illumination, imaging, as well as the movement and control units, we have created a system that is able to efficiently acquire line scan profiles of a sample and perform a rapid quantitative 3D surface microstructure reconstruction based on the laser trigonometry method [28]. To avoid complex calculations, an orthogonal geometrical light path is devised to derive a linear calibration relationship between surface height/depth and pixel offset. Additionally, the simple mechanical structure makes it easy to exchange objectives with different magnification, meeting variations in demands of corresponding resolution or FOV.

## 2. System Setup

**Figure 1:**
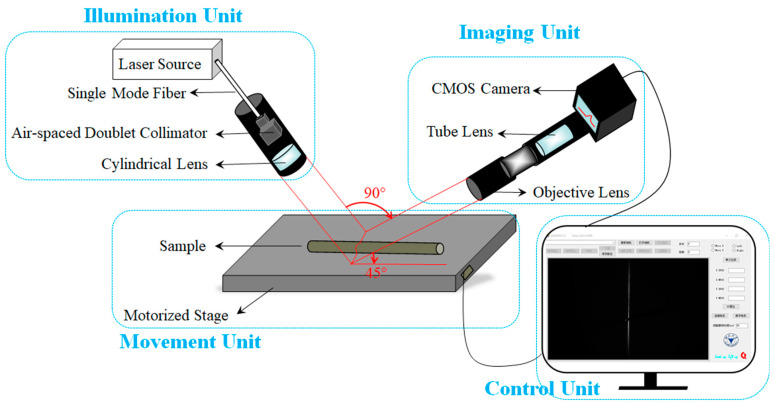
Schematic diagram of the light-sheet microscopy (LSM) system, which consists of four parts: an illumination unit, an imaging unit, a movement unit and a control unit, the details of which are described in Section 2.

Our LSM system consists of an illumination unit, an imaging unit, a movement unit and a control unit, as depicted in Figure 1. The illumination unit uses a 785 nm semiconductor laser (Laser-785, Ocean Optics, Largo, FL, USA) as the light source that is coupled to a single-mode optical fiber and outputted to an air-spaced doublet collimator (F810SMA-780, Thorlabs, Newton, NJ, USA). The air-spaced doublet collimator is chosen to match the characteristics of the laser source in order to optimize the collimation, which means it can output the light with excellent signal-to-noise ratio (SNR). The illumination spot is compressed by a cylindrical lens (f = 25 mm, DHC, Beijing, China), such that the light-sheet is at its thinnest in the focal plane of the cylindrical lens. This approach, to use a cylindrical lens to generate a light-sheet in the illumination unit, is simple and straightforward as compared to, e.g., obtaining a virtual light-sheet using galvomirror scanning, as done in high-resolution DSLM [19,20]. The imaging unit consists of a 4× microscope objective (UPlanFLN, NA = 0.1, Olympus, Tokyo, Japan), a tube lens (f = 50 mm, DHC, Beijing, China) used to create an infinite imaging space, and a CMOS camera (ASI174MM, ZWO, Suzhou, China) for image capture. Under the 4× objective, a large FOV for observing and imaging can be achieved. The illumination and imaging units are arranged perpendicular to each other and at 45° angles to the horizontal plane to facilitate the calculation of height/depth in the *z*-direction (see discussion in the following section). The movement unit is an XYZ three-axis movable displacement stage (HDS-CBMS-XYZ-I-R, HEIDSTAR, Xiamen, China) used for adjusting the focal plane in the *z*-direction as well as for the line scanning of the sample in the x or y direction. The computer control unit controls the displacement of the stage, the CMOS camera, as well as performing stitching reconstruction of collected data. Control software is written in C# and surface topography reconstruction routines are written in MATLAB. Using the control software interface (shown as the control unit in Figure 1), the exposure time and gain can be set according to the reflectivity of a diversity of samples, ranging from 32 μs to 5 s and 1 to 400, respectively. The scanning direction, step and range, can as well be set based on the demands of the application, with a minimum step of 10 μm and a maximum moving range of 80 mm. The control interface also provides a function for real-time previsualization.

## 3. Laser Triangulation and System Calibration

### 3.1. Laser Triangulation

**Figure 2:**
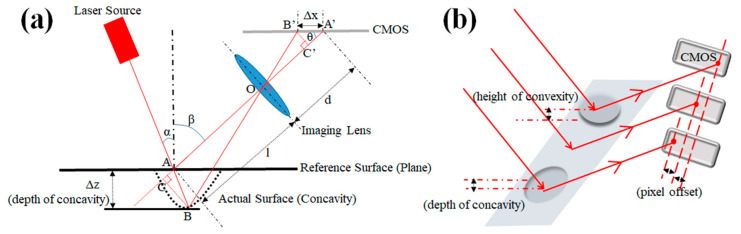
(**a**) Schematic diagram of the universal oblique laser triangulation. The reference surface refers to the plane of the sample to be inspected were it to be an ideal flat surface as opposed to the actual surface including surface imperfections. We refer to concavities or convexities for cases when the actual surface is below or above the reference surface (the dotted line in the figure indicates a concavity). O is the origin of the lens plane, A is a point on the reference surface, B is a point on the actual surface. Both A and B are illuminated by the light-sheet, and A′ and B′ are their respective images. *α* is the angle between the incident light and the surface normal, *β* is the angle between the elastically scattered light and the surface normal. *l* is the distance from object point A to the imaging lens and d is the image distance, which satisfies the lens equation. Δ*z* is the depth/height of a concavity/convexity and Δ*x* is the length of A′B′ in the CMOS plane. *θ* is the angle between AA′ and the plane of the CMOS camera. *f* is the focal length of the imaging lens. In our system, both *α* and *β* are set to 45°, and *θ* is set to 90°, as discussed below. (**b**) Schematic illustration of the relationship between the depth/height of a concavity/convexity and the pixel offset in the CMOS camera. A concavity/convexity in the surface corresponds to a pixel shift to the left/right.

The principle of optical non-contact triangulation is the basis for the quantitative calculation in our LSM system. Figure 2 shows the optical path of the oblique laser triangulation. The light-sheet, whose illumination plane is perpendicular to the page, laterally illuminates a portion of the sample, and the elastically scattered light received by the imaging lens is collected by the CMOS camera. The light-sheet has a thickness of about 50 μm.

Using the definitions in Figure 2 and the apparent fact that the triangles OCB and O′C′B′ are similar, we find

(1) $$\frac{\left|\mathrm{BC}\right|}{\left|\mathrm{OC}\right|}=\frac{\left|B^{\prime }C^{\prime }\right|}{\left|{\mathrm{OC}}^{\prime }\right|}$$

(2) $$\frac{\left|\mathrm{AB}\right|\sin\left(\alpha +\beta \right)}{l+\left|\mathrm{AB}\right|\cos\left(\alpha +\beta \right)}=\frac{\Delta x\sin\theta }{d-\Delta x\cos\theta }$$

(3) |AB|cosα=Δz

With *f* being the focal length of the imaging lens, the lens equation reads

(4) $$\frac{1}{l}+\frac{1}{d}=\frac{1}{f}$$

Combining Equations (1)–(4), we obtain the relationship between the offset Δ*z* of the surface relative to the reference plane in the *z*-direction and the offset length Δ*x* in the plane of the CMOS camera to be

(5) $$\Delta z=\frac{\Delta x\sin\theta \cos\alpha \left(l-f\right)}{f\sin\left(\alpha +\beta \right)\pm \Delta x\sin\left(\alpha +\beta +\theta \right)\left(1-f/l\right)}$$

Sign ± in Equation (5) refers to when the actual surface is above or below the reference plane (convex or concave surface cases). Following the system setup, *α*, *β*, *θ*, *f* and *l* are all fixed so the offset length Δ*x* in the plane of the CMOS camera can be calculated to correspond to the variation Δ*z* of the sample. With the angles set to be *α* = *β* = 45° and *θ* = 90°, Equation (5) reduces to

(6) $$\Delta z=\frac{\left(l-f\right)}{\sqrt{2}f}\Delta x$$

i.e., we find Δ*z* to be linearly proportional to Δ*x*, which is consistent with the results of Xia et. al. [2]. Thus, choosing a system setup such that the illumination unit and the imaging unit are perpendicular to each other and at 45 degrees from the reference plane greatly simplifies the calculations. It should be mentioned that the system in Reference [2] uses a slit to generate the light-sheet, which causes significant energy loss. In contrast, we produce the light-sheet by compressing collimated light spot through a cylindrical lens so that the utilization of laser source is more efficient. We also create an infinite imaging space by using a tube lens, removing the need for an eyepiece, which is required in the system in Reference [2]. The geometrical model we utilize is also convenient and legible, as opposed to the complex light tracing based on the ABCD law utilized in Reference [2].

In summary, irregularities in the sample surface will modulate the shape of the light-sheet, which, in turn, is registered by the CMOS camera, and the magnitude of the surface irregularity can be calculated by the correspondence relationship (6) derived above.

### 3.2. System Calibration

**Figure 3:**
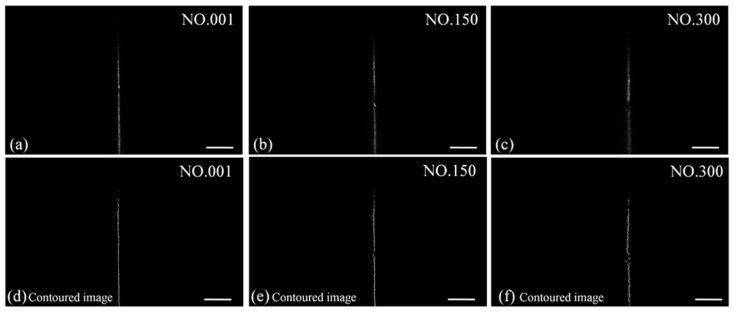
Captured images of the light-sheet during *z*-direction offset calibration. Images from the 1st, 150th and 300th steps are shown in (**a**–**c**). After contour extraction, the contoured images are shown in (**d**–**f**). Scale bar: 1 mm.

The fact that our ′imaging lens′ in actuality consists of the combination of a microscope objective and a tube lens necessitates a calibration to derive a quantitative relationship between Δ*x* and Δ*z*. This is done as follows: let the light-sheet illuminate the surface of the displacement stage, which initially is located in the focal plane of the imaging unit, and adjust the illumination unit so as to minimize the beam waist of the light-sheet. Following this, the displacement stage is moved upwards in increments of 2.5 μm, and at each step the vertical lines are recorded on the image surface of the CMOS camera. As the plane of the light-sheet moves upwards, the vertical lines collected on the image surface of the CMOS camera will gradually shift to the right. A total of 301 images are collected in the calibration, some of which are shown in Figure 3a–c.

Due to the good coherence characteristics of the laser source, there is an inherent speckle noise in the images. In order to eliminate this, a MATLAB-algorithm is used to extract the profile of the light-sheet, the steps of which are the following:

1. Load the original image into the MATLAB workspace and convert the image to a gray matrix named ′Img′ (1280 × 1936);
2. Apply MATLAB function ′imguidedfilter′ to perform image-smoothing, and remove part of the laser speckle;
3. Loop i from 1 to 1280, for each horizontal line, then create a vector hor_prol = Img(i,:), that represents the intensity line profile;
4. Loop j from 1 to 1936, to find the maximum value of hor_prol(j) and denote j as the brightest (most intense) position of this line. Due to the limitation of the image bits, the maximum value of hor_prol(j) may appear in multiple positions (i.e., more than one brightest point in a row). If there is more than one hor_prol(j), the corresponding to maximum value, take all satisfied j as the average j, then break the loop. Img(i,j) means the brightest position of i row.

**Figure 4:**
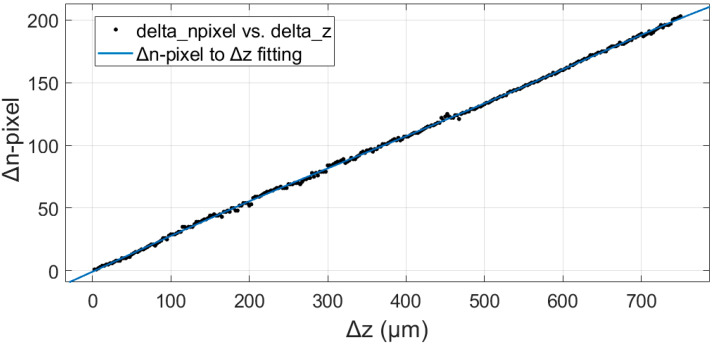
The fitted relationship between the pixel offset in the CMOS camera, Δ*n*_pixel_, and the displacement in the *z*-direction, Δ*z*.

Using this algorithm, an equivalent set of contour images can be obtained. Figure 3d–f shows the equivalent contour images to the captured light-sheet images shown in Figure 3a–c. By subtracting the horizontal value of the pixel location of each of the 301 recorded contour lines with that of the first contour line, we obtain 300 Δ*n*_pixel_-values. These are defined as the equivalent to the physical offset length Δ*x* in the surface plane of the CMOS camera such that

(7) $$\Delta x={\Delta n}_{\mathrm{pixel}}\times {\mathrm{size}}_{\mathrm{pixel}}$$

where size_pixel_ is the size of the pixels in μm. We can then derive a relationship between Δ*n*_pixel_ and Δ*z* that is equivalent to Equation (6) by fitting a 5th-order polynomial, the result of which is plotted in Figure 4.

The fitting formula is as follows:

(8) $${\Delta n}_{\mathrm{pixel}}=-6.26e^{-13}{\Delta z}^{5}+1.06e^{-9}{\Delta z}^{4}-5.33e^{-7}{\Delta z}^{3}+4.52e^{-5}{\Delta z}^{2}+0.29\Delta z-0.74$$

The R-square value of the fit is 0.9996. From the graph in Figure 4 and the fitting relationship, we see that the high-order terms of Δ*z* are several orders of magnitude smaller than the first-order term and can safely be ignored. The fitting formula quite clearly verifies the linear relationship of the surface height variations and the pixel offset in the CMOS camera, as predicted by the laser triangulation method in Section 3.1. The fitting of Formula (8) forms the basis for the quantitative calculation of the surface height variation in the samples in our experiments. 

Concerning system resolution, we note that, due to the influence of speckle noise and the uniformity of the light-sheet, a change Δ*z* may occasionally yield Δ*n*_pixel_ = 0, i.e., the resolution of the system on the *z*-axis is larger than Δ*z* = 2.5 μm. Moving up to 4 Δ*z*, then Δ*n*_pixel_ must be greater than 1, indicating that the limit of the system resolution in the *z*-axis direction is at the most at 4 Δ*z* = 10 μm.

**Figure 5:**
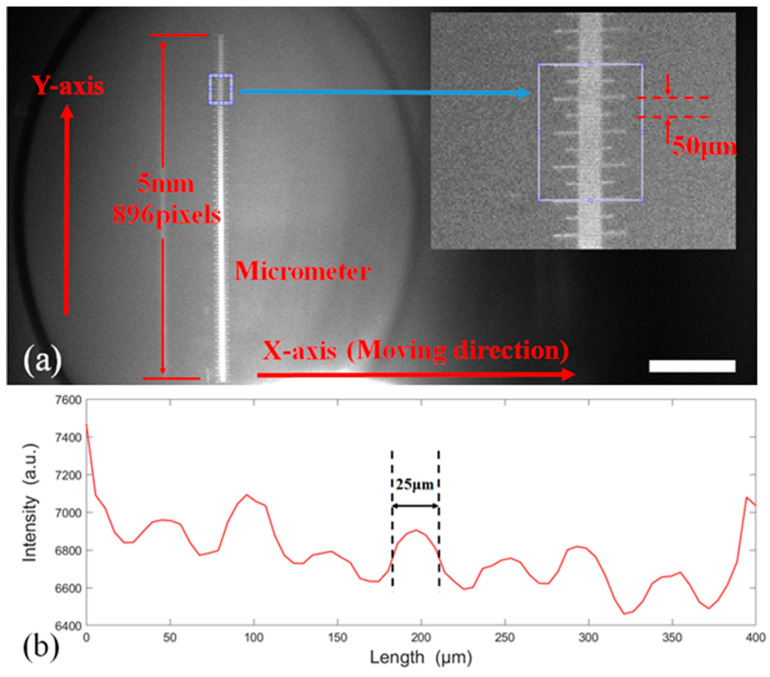
(**a**) Micrometer ruler used to determine the system resolution in the *y*-direction. Insert shows a magnification of the section used in the measurement. Scale bar: 1 mm. (**b**) Calculated line profile of the optical intensity.

To estimate the resolution in the *y*-direction. we evaluate a micrometer ruler that has line engravings spaced at 50 μm, as shown in Figure 5a. The number of pixels in the *y*-direction of the CMOS camera is proportional to the spatial length, meaning that 896 pixels (the total number of pixels) correspond to 5 mm (the full length of the imaged area). For a selected a part of the micrometer ruler, we calculate the line profile of the optical intensity part in the vertical direction, shown in Figure 5b. By calculating the full width at half maxima (FWHM) of the intensity peaks. we can estimate the spatial resolution in the *y*-direction to be 25 μm, which is accurate enough for a wide range of applications. 

In the *x*-direction, i.e., the direction of the motorized stage scanning, the resolution is directly dependent on the minimum step length of motorized stage, which in our system is 10 μm.

## 4. Test Measurements

In this section, we describe the measurements done to test and verify the functionality of our LSM system. These are done on a single strand of hair, stacked feeler gauges, a solar cell panel and a mobile phone protective shell.

### 4.1. Measurement of the Diameter of a Single Strand of Hair

**Figure 6:**
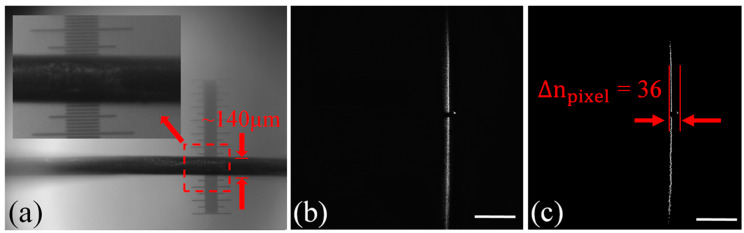
Measurement of the diameter of a single strand of hair at one position. (**a**) Strand of hair placed on micrometer ruler with a resolution of 10 μm. (**b**) Measured contoured image of the strand of hair. (**c**) Enlarged view of (**b**) to indicate pixel offset Δ*n*_pixel_ = 36 due to the light-sheet modulation. Scale bar: 1 mm.

For a single strand of hair, we measure its diameter at the same location with both our LSM system and a micrometer ruler. First, we place the strand of hair on a micrometer ruler whose resolution is 10 μm, as shown in Figure 6a, and find, through optical inspection using a 10× microscope objective, its diameter to be about 140 μm. Next, we measure the diameter of the strand of hair at the same location as a surface height variation using our LSM system. The strand of hair is irradiated by the light-sheet as shown in Figure 6b, where we can clearly see a modulation of the light-sheet produced by the strand of hair. We record a picture of the light-sheet irradiating the strand of hair and, after contour extraction, we find the pixel offset Δ*n*_pixel_, to be 36, which, according to Equation (6), corresponds to Δ*z* = 141 μm. This agrees well with the value of 140 μm measured directly with the micrometer ruler of 10 μm resolution.

### 4.2. Measurement of Height Variations of Stacked Feeler Gauges

**Figure 7:**
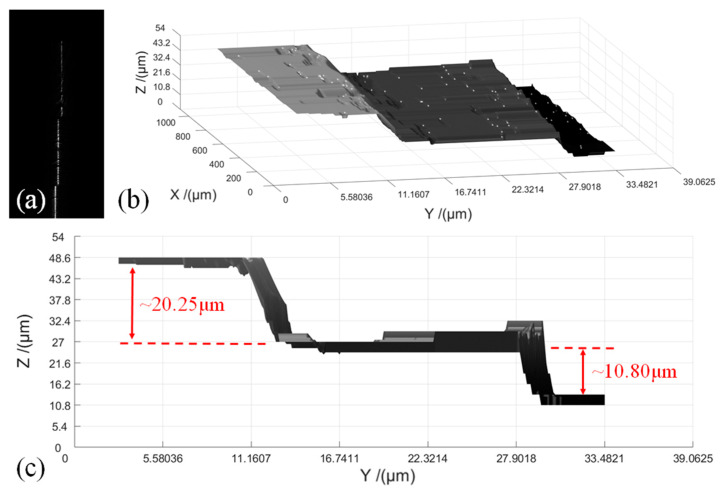
Measurement of height variations of two feeler gauges stacked in a stair shape. (**a**) Light-sheet modulated by two feeler gauges. (**b**) Calculated 3D topography of the feeler gauges. (**c**) Side view of the ′stair′.

To demonstrate the usability of our LSM system for the inspection of micro-meter sized samples, we measure the height variations in two feeler gauges with thicknesses of 10 and 20 μm that are stacked in a stair shape. The feeler gauges are illuminated vertically, and the modulated light-sheet is shown in Figure 7a. A total of 100 sectional images are acquired at an interval of 10 μm, with a total scan time of about 20~30 s. The continuous scanning images are subsequently processed using MATLAB to be stitched into a full 3D topography, which is shown in Figure 7b. The 3D reconstruction takes less than 5 s, and the reconstruction algorithm is shown as follows:

1. Use ′cat′ function to generate a point clouds model;
2. Interpolate scattered 3D data using ′griddata′ function;
3. Create a 3D surface plot utilizing ′surf′ function.

Figure 7c shows a side view of the 3D topography, where the steps of the stacked feeler gauges are clearly seen. The height of the two feeler gauges are calculated to be 20.25 μm and 10.80 μm, with relative errors to the actual height of the feeler gauges of 1.25% and 8.00%. Considering the acceptable range of errors for most application areas for this system, we conclude that our LSM system is sufficiently accurate for quantitative inspection of surface variations in the *z*-direction.

### 4.3. Surface Topography Inspection of a Solar Cell Panel

**Figure 8:**
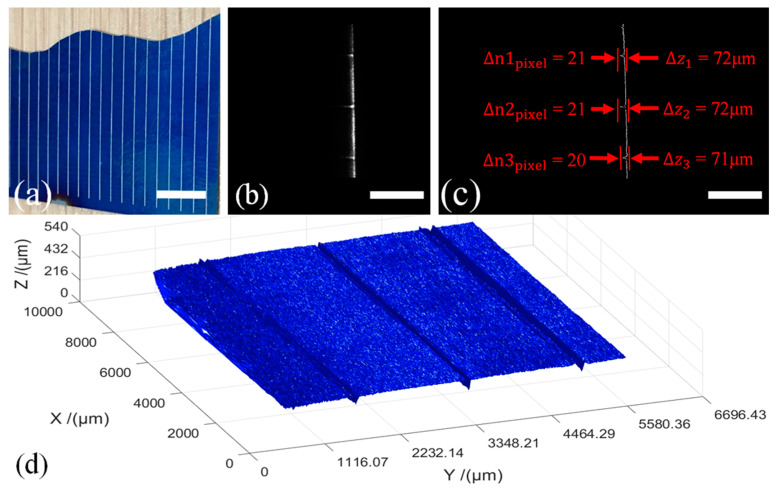
Surface topography inspection of a solar cell panel. (**a**) The solar cell panel used in the experiment. Gate lines are the white lines. Scale bar: 6 mm. (**b**) Light-sheet modulated by the gate lines. Scale bar: 1 mm. (**c**) Measured contoured image. Scale bar: 1 mm. (**d**) Calculated 3D surface topography of the solar cell panel.

In this measurement, we inspect the surface of a solar cell panel on which there exist parallel gate lines, as shown in Figure 8a. The depth of the gate lines is a relevant index of the solar cell′s quality as pertaining to power generation efficiency and device lifetime. We obtain a total of 182 sectional images at an interval of 50 μm during 60 s. A light-sheet, modulated by the gate lines, is shown in Figure 8b, the results of the contour extraction and the profile of the first light-sheet are shown in Figure 8c, and the calculated full 3D surface topography is shown in Figure 8d. The reconstruction takes less than 20 s. We choose three gate lines in the measurement range to make sample depth calculations. First, we determine the pixel offset of the indentures of each picture profile relative to the vertical reference line, after which we calculate the average value Δ*n*_pixel_ of the 182 picture pixel offsets. These, we find to be Δ*n*_pixel_ = 21, 21 and 20, respectively, and using Equation (8) we find the depth of the three gate lines to be 72, 72 and 71 μm, respectively.

To verify the accuracy of the LSM system measurement, we use a stylus profiler (Veeco Dektak 150, NJ, USA) to measure the gate line depth. The measured result is 74.5 μm from which we calculate the relative errors of the LSM system depth measurements to be 3.4%, 3.4% and 4.7%, respectively, indicating sufficient accuracy of LSM system measurement.

This sample measurement demonstrates the ability of our LSM system to inspect microstructures on industrial sample surfaces.

### 4.4. Surface Topography Measurement and Curvature Calculation of an Opaque Mobile Phone Protective Shell

**Figure 9:**
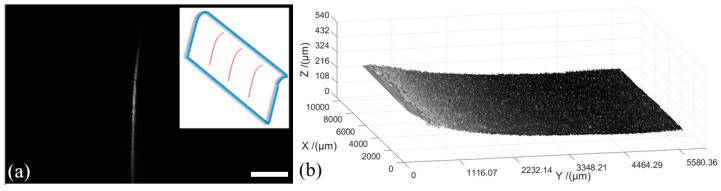
Surface topography measurement and curvature calculation of an opaque mobile phone protective shell. (**a**) The curved side of the shell irradiated by the light-sheet. Scale bar: 1 mm. (**b**) Calculated 3D surface topography of the phone shell.

Finally, we perform a scanning measurement of an opaque mobile phone protective shell that has a curved edge. The curved edge of the shell is vertically irradiated by the light-sheet, as indicated in Figure 9a. We obtain a total of 201 sectional images taken at an interval of 50 μm. Using contour extraction, the 3D surface topography map is calculated as shown in Figure 9b.

**Figure 10:**
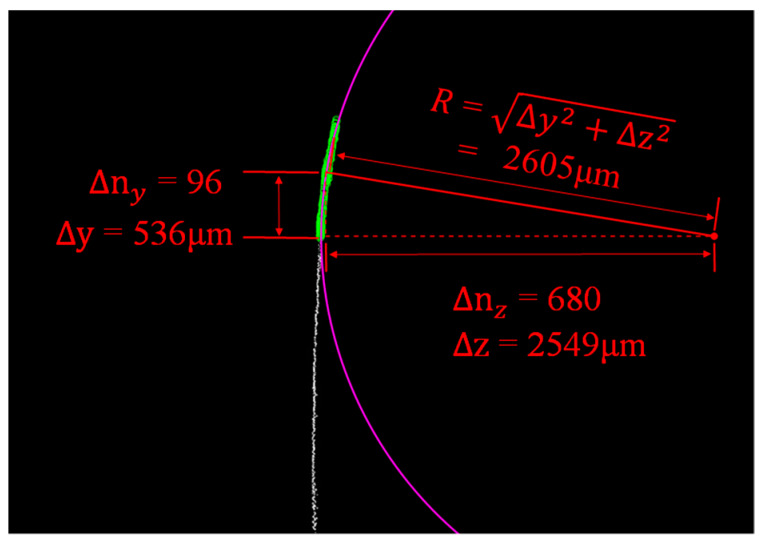
Calculation of the curvature of the opaque mobile phone protective shell through fitting to a circle. The circle radius is calculated by recognizing that Δ*y*/Δ*n_y_* and Δ*z*/Δ*n_z_* are the projections of the radius in the *y*- and *z*-directions, and thus R is found using Pythagoras′ theorem.

Using one light-sheet profile as an example, we demonstrate that the curvature of the shell side can be calculated. The contour image is shown in Figure 10 together with a circular fitting curve. For the calculation, we consider only the curved section of the contour image (labeled green in Figure 10) and find the radius of the fitting circle to be R = 2605 μm ≈ 2.6 mm, i.e., the curvature of the phone shell side is 1/2.6 = 0.38/mm (details in Figure 10 and caption).

This measurement shows that our system can be utilized to calculate sample parameters that are outside the surface plane and suggests quality inspection of industrial pipelines as a potential application.

## 5. Conclusions and Outlook

Based on line-scanning technology and the laser triangulation method, we have developed a light-sheet microscopy (LSM) system that uses reflected/scattered light to quantitatively inspect surface variations in opaque samples. A linear relationship between the surface height variations and the pixel offset in the CMOS camera enables convenient system calibration. The resolution in the *z*-direction of the LSM system presented here is better than 10 μm. The resolution is 25 μm in the *y*-direction, however, higher resolutions can easily be obtained by replacing the objective used in the current setup with an objective with higher magnification, and the system has a simple mechanical structure that allows for easy part replacement. The resolution in the *x*-direction is 10 μm and can be improved by reducing the minimum step length of the motorized stage.

The sample measurements in Section 4 demonstrate both the functionality of our LSM system as well as indicating possible areas of application, such as accurate measurements of structural dimensions, surface defect inspection, microstructure imaging, and topography analysis of opaque objects, making this a promising and versatile tool for industrial sample inspection. Extending this to longer light-sheets and a larger scanning range by increasing the size of some of the system components would further increase the potential uses. We may additionally combine the LSM system with adaptive optics to eliminate aberrations [29]. Furthermore, the position of the object plane and imaging plane can be adjusted to intersect the lens plane on the same line, whose position relationship fits the Scheimpflug principle [30]. In this case, an entire plane can be imaged in focus and we could thus acquire clear images of the entire plane [31]. 

In terms of future improvements, some limitations of the current systems should be mentioned. Due to the focal length of the cylindrical lens, the diffraction limit affects the contour. In addition, when performing straight-line profile extraction, the existing algorithm is affected by the influence of speckle noise that may cause errors in the extracted line, as discussed in Section 3.2. More advanced algorithms trained on a large number of datasets could be a route to realize more accurate calibration and pixel offset calculations. 

By combining our LSM system with a phase contrast imaging method, the method may be utilized for 3D imaging of transparent samples, such as a microlens array. Such a system could also be utilized for the imaging of living biological samples due to its excellent imaging speed and low phototoxicity and low photobleaching. We furthermore notice that, as our system utilizes reflected/scattered light for 3D optical-sectioning measurements at the micron level, 3D surface analysis of translucent and layered sample is possible.

In summary, we have developed a novel LSM system that can be used for the quantitative calculation of the microstructure topography that has great potential for use in a wide range of applications for, e.g., industrial inspection and biological sample analysis.
